# Distribution and variability of total mercury in snow cover—a case study from a semi-urban site in Poznań, Poland

**DOI:** 10.1007/s11356-016-7627-8

**Published:** 2016-09-21

**Authors:** Patrycja Siudek

**Affiliations:** Department of Water and Soil Analysis, Faculty of Chemistry, Adam Mickiewicz University in Poznań, Umultowska Street 89b, 61-614 Poznań, Poland

**Keywords:** Mercury, Snow cover, Urban, Anthropogenic, Emission

## Abstract

In the present paper, the inter-seasonal Hg variability in snow cover was examined based on multivariate statistical analysis of chemical and meteorological data. Samples of freshly fallen snow cover were collected at the semi-urban site in Poznań (central Poland), during 3-month field measurements in winter 2013. It was showed that concentrations of atmospherically deposited Hg were highly variable in snow cover, from 0.43 to 12.5 ng L^−1^, with a mean value of 4.62 ng L^−1^. The highest Hg concentration in snow cover coincided with local intensification of fossil fuel burning, indicating large contribution from various anthropogenic sources such as commercial and domestic heating, power generation plants, and traffic-related pollution. Moreover, the variability of Hg in collected snow samples was associated with long-range transport of pollutants, nocturnal inversion layer, low boundary layer height, and relatively low air temperature. For three snow episodes, Hg concentration in snow cover was attributed to southerly advection, suggesting significant contribution from the highly polluted region of Poland (Upper Silesia) and major European industrial hotspots. However, the peak Hg concentration was measured in samples collected during predominant N to NE advection of polluted air masses and after a relatively longer period without precipitation. Such significant contribution to the higher Hg accumulation in snow cover was associated with intensive emission from anthropogenic sources (coal combustion) and atmospheric conditions in this area. These results suggest that further measurements are needed to determine how the Hg transformation paths in snow cover change in response to longer/shorter duration of snow cover occurrence and to determine the interactions between mercury and absorbing carbonaceous aerosols in the light of climate change.

## Introduction

Urban snow is a key parameter in hydrological, meteorological, and ecological non-linear processes in the environment (Engelhard et al. 2007). Mercury, as a highly toxic contaminant, can reinforce its negative impact on biotic and abiotic processes in aquatic and terrestrial ecosystems after snowmelt (Wiener et al. 2003). In-situ long-term measurements of gaseous elemental Hg (GEM) and its oxidized species (RGM, reactive gaseous mercury: HgP, particulate mercury) in the snowpack and overlaying atmosphere have been intensively undertaken over the past decade by various polar research groups in the Canadian Arctic, Antarctica, and Greenland. The unique phenomenon called atmospheric mercury depletion event (AMDE) is very promising scientific issue with regard to further researches on Hg atmospheric chemistry in polar regions (Lu et al. 2001, Witherow and Lyons 2008). It was observed that GEM can be removed during springtime from lower atmosphere via photochemical processes and rapidly deposited on the snow cover, resulting in extremely low values of GEM above the polar snowpack. Recent studies at polar sunrise revealed that total mercury concentrations in the surface snowpack are elevated, up to 100 ng L^−1^ (Lahoutifard et al. 2005, Larose et al. 2013), compared to other non-polar locations.

Despite clear evidence for Hg emission from the snowpack at high latitudes, similar mechanism in relation to lower latitudes (Europe and Northern America) has several uncertainties. Data on vertical distribution of Hg and its seasonal variability in the snow cover from non-polar regions are limited. However, this subject has been investigated in Europe recently, for example, Siudek et al. (2014) observed a very irregular distribution pattern of Hg in the urban snowpack from Gdynia (northern Poland), with a maximum Hg concentration of 22.5 ng L^−1^. Measurements of Hg by Nelson et al. (2007) showed that intensive coal combustion in residential and commercial buildings during winter period caused high Hg concentrations in the snowpack from Maine, US. Faïn et al. (2007) and Ferrari et al. (2002) also found extremely high amount of mercury in alpine snow samples, suggesting the increasing trend in anthropogenic Hg emission during cold season. Mercury emission to the atmosphere during the heating season significantly affects snow cover properties and composition. The influence of urban environments on Hg processes in the air, water, and sediments is of crucial interest and depends on many factors such as site characteristics, emission rate, distance to anthropogenic sources, and meteorological background. (De Simone et al. 2014, Li et al. 2015, Liu et al. 2015, Siudek et al. 2015a). Although the main sources of Hg in the snowpack are well determined, the quantitative analysis of Hg in snow cover system may be of crucial importance to study processes such as transfer of Hg species from the overlaying air to snow and possible re-emission or retention of Hg, and to better understand the impact of small-scale dynamics of Hg on the environment and air quality.

The present study was based on eight snow experiments carried out in an urban area of central Poland, in one winter season. The experiments were conducted to identify how particular factors connected with atmospheric conditions, i.e., air temperature, wind speed and direction, impact of local/regional, and remote emission sources, can influence retention of atmospheric mercury in shallow snow cover and to describe the role of Hg transformation processes inside the snow column. To achieve these aims, a combination of field measurements, chemometric techniques (PCA), and backward trajectory analysis (HYSPLIT model) was implemented. The role of other inorganic pollutants measured in snow cover was also considered. Therefore, the results from these experiments can be used for further modeling studies and chemical calculations to estimate the impact of Hg deposition with snow on the ecosystem of similar regions.

## Materials and Methods

### Study area

The city of Poznań, as an example of inland urban area, has been chosen for the present study on mercury in snow cover. Poznań (city area 261.8 km^2^, inhabitants of up to 600,000) is located in the Wielkopolska Province, central Poland. It is influenced by various industrial and urban emission sources (diurnal and/or seasonal time scale). Main Hg sources include the following: coal-burning heat and power plants, individual power generating stations and domestic furnaces where coal is the main fossil fuel, residential and domestic buildings, dumping grounds for municipal wastes, domestic sewage, cement factories, sewage treatment plants, airport infrastructure, transportation, different industrial units producing metals and paints, and high-temperature industrial processes (smelting, waste incineration).

The climate in central Poland is temperate, with annual mean temperature of about 8.5 °C, frequent precipitation events, and prevailing wind direction from the northwest. Poznań has the lowest yearly sum of precipitation in Poland (<550 mm) and 32 % of falls occurs during winter season. In this region, the number of days with snow cover and air temperatures below 0 °C is relatively low as compared with high mountains or poles. Snow cover typically occurs between November and late March. Its composition is strongly affected by atmospheric conditions (advection of polluted air masses, city heat island), snowpack processes (compaction, heat conduction, turbulent fluxes), soil and air temperature, and liquid water content. Thus, the occurrence and amount of snow may significantly vary both spatially and temporally, even within a single winter season. In the present study, the average time for individual snow cover (min. 5 cm) formation ranged from 10 to 24 h and depended highly on specific meteorological patterns. Table 1 presents data for each snow event and its meteorological background. Snow experiments were divided according to the month of sampling, i.e., J—snow experiments that were performed in January (two case studies), F—snow samples collected in February (three case studies), and M—snow cover sampled in March (three case studies). Such classification has been applied to indicate major factors (seasonal and meteorological) influencing Hg variability in snow cover.

**Table 1:** Statistical characteristics of atmospheric conditions in Poznań during the sampling days in winter 2013

Code	Date	Snow cover depth (cm)	Number of snow layers	Mean temperature ± SD (min–max)	Mean relative humidity ± SD (min–max)	Mean pressure ± SD (min–max)	Mean wind speed ± SD (min–max)	Prevailing wind direction
J1	25/01	15	11	−6.6 ± 4.0	93 ± 8.5	1011 ± 1.2	0.2 ± 0.3	N, NE
				−12.4 to −0.2	75–95	(1008–1012)	(0.1–1.1)	
J2	28/01	10	9	−2.9 ± 2.2	97 ± 1.6	1001 ± 3.4	1 ± 0.3	NW, W
				−6 to −0.2	92–98	(996–1005)	(0.6–2)	
F1	13/02	6	5	1.1 ± 1.4	89 ± 11.2	1004 ± 0.9	0.7 ± 0.2	S, SE
				−0.7 to 3.3	70–98	(1002–1005)	(0.5–1.4)	
F2	14/02	10	10	−0.4 ± 0.9	98 ± 0.2	1014 ± 1.9	0.3 ± 0.3	SE, E
				−1.9 to 3.4	97–98	(1011–1016)	(0.2–1.1)	
F3	19/02	12	11	−0.5 ± 0.5	98 ± 1.0	1010 ± 2.9	1.4 ± 0.5	NW, W
				−1.2 to 1.6	94–98	(1004–1014)	(0.6–2.2)	
M1	11/03	20	13	−2.9 ± 0.6	90 ± 6.8	995 ± 2.0	0.2 ± 0.7	NE, E, SE
				−3.8 to −2.0	81–98	(991–997)	(0.1–2.4)	
M2	19/03	25	16	0.1 ± 3.1	56 ± 10.0	1006 ± 2.7	2.5 ± 0.7	S, SE
				−4.7 to 4.5	43–74	(1002–1010)	(1.1–3.7)	
M3	25/03	20	14	−3.3 ± 5.9	73 ± 17.4	1016 ± 0.9	0.5 ± 0.4	NE, E
				−13.7 to 2.2	50–98	(1015–1018)	(0.1–1.5)	

### Sampling method

**Fig. 1:**
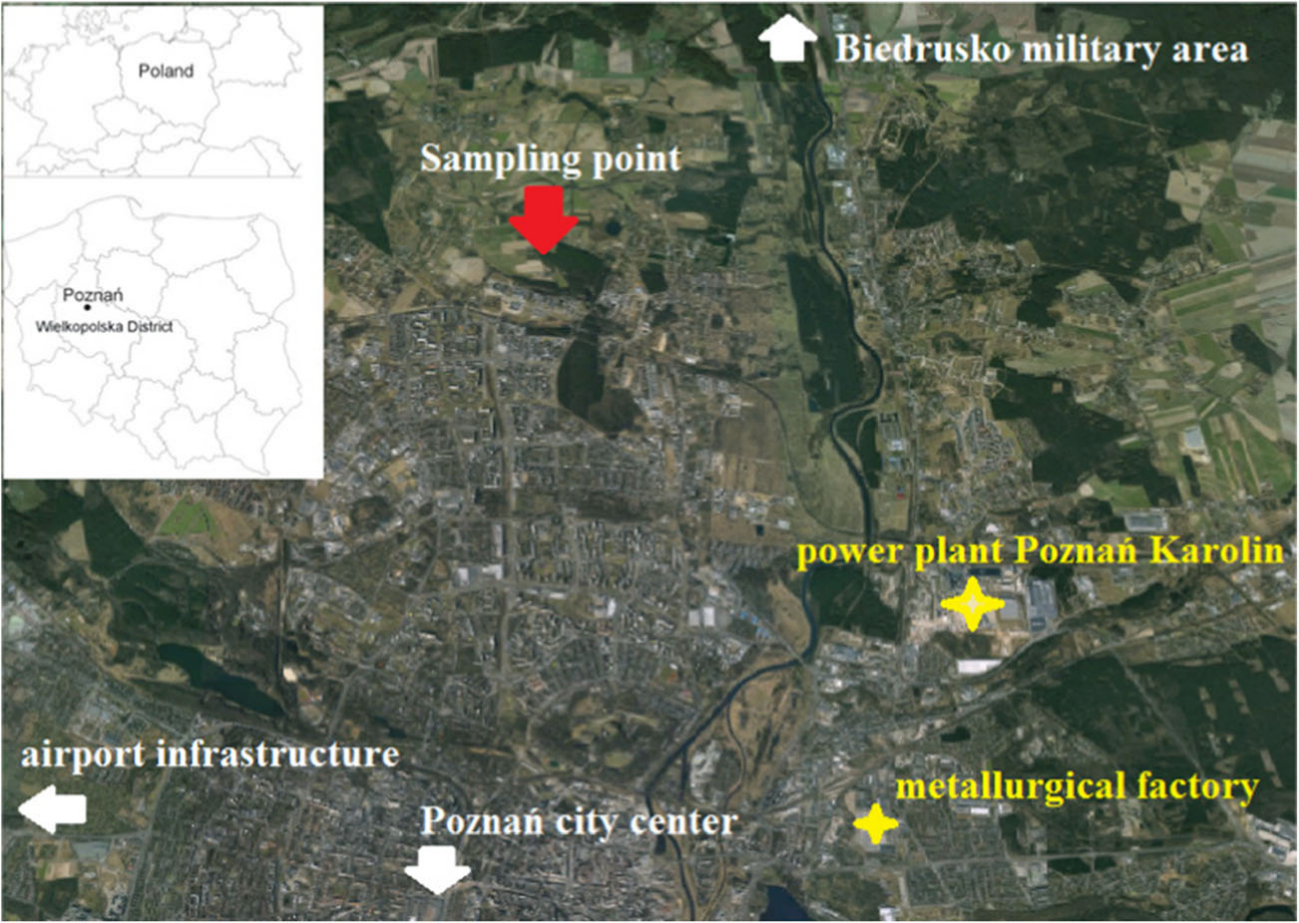
Map of the sampling site—a semi-urban area, Poznań, Poland

Snow experiments were conducted at the semi-urban site (52° 42′ N, 16° 88′ E), in January, February, and March, 2013. The sampling site, located at the Adam Mickiewicz University campus in Morasko, is approximately 10 km north of Poznań city center (Fig. 1). Previous aerosol-related measurements in this area provided a synthetic report of most significant local and regional trace metal emission sources, i.e., fuel combustion, industrial processes, and heavy traffic (Siudek et al. 2015b, 2016). The surrounding area located to the southeast from the study domain is industrially impacted and significantly contributes to air pollution, while the northern sector represents mainly forests, rural, and sub-urban areas. It has been indicated that various anthropogenic sources have the impact on air quality in Poznań and adjacent areas; however, in winter, coal combustion in local domestic heating units (DHUs) plays a predominant role. Large Hg point sources are located within 10–20 km from the sampling site, e.g., CFFPs Poznań Karolin and metallurgical factory—about 5 km to the southeast; residential boilers, waste incinerations, Biedrusko military area—about 10 km to the north; rail freight transport and roads—about 1 km to the south.

To obtain highly representative data, special precautions were undertaken. We selected a flat terrain, far from buildings, with several trees around. A special open system (1 × 1 × 0.3 m PTEF-coated cuvette) was installed to prevent snow cover from possible negative artifacts, i.e., from dust deposition on freshly fallen snow cover, diffusion and wind pumping effects, snow drifting, or snow blowing. Samples were taken after each snow event, within no longer than 12 h of snow cover formation. A full set of meteorological and physical measurements was performed at the sampling site. Specifically, we measured snow cover depth through the use of a polyethylene stick. The key step in our sampling method was to perform in-situ separation of snow layers. Each snowpack was divided into several subsamples (1-, 2-, 3-cm layers). Snow layers were carefully separated from the whole column (starting from the snow surface, i.e., snow-air interface) and placed in pre-cleaned bottles. Then, all samples were transported to the laboratory. Snow sublayers were separated through the use of a pre-cleaned Plexiglas device and plate. Acid-cleaned borosilicate glass bottles and powder-free gloves were used to avoid sample contamination. A more detailed description of the adopted method can be found in Siudek et al. (2014). In the present study, we collected samples from at least 5-cm snow cover, for which the in-situ separation of snow layer was much easy and therefore provide better accuracy of Hg determination.

After snow melting (in closed bottles), 30 ml of a non-filtered aliquot (homogenized by shaking) was acidified with trace element grade HNO_3_ (0.5 % *v*/*v*, Sigma Aldrich) to pH < 2 and stored for less than 2 weeks to the main analysis. The applied procedure (without filtration) ensures minimal loss of Hg species in snow samples. Lab glassware used in all snow experiments (bottles, plates) was rigorously acid-soaked in 3 M HNO_3_ for 1 week and then rinsed 5 times with double distilled water (DDW), dried under laminar flow in a clean bench, and stored in polyethylene bags.

Total Hg concentrations (dissolved and particulate fractions) in melted and non-filtered snow samples were analyzed by cold vapor atomic fluorescence spectrometry (CVAFS) on PSA Millennium Merlin mercury analyzer (UK), according to the standard method for Hg (US EPA method 1631 2002). In general, the above-mentioned analytical procedure is based on gold amalgamation and atomic fluorescence detection. Prior to the sample analysis, all Hg species were oxidized with BrCl, neutralized with NH_2_OH·HCl solution, and then reduced with SnCl_2_. Laboratory analysis of bottles (every 10 rigorously acid-washed borosilicate glass bottles) and field blanks (snow corer rinsed with DDW) was performed prior to the main analysis. The bottle blanks were processed in parallel to the environmental samples, and their mean Hg values were below the detection limit, indicating that no contamination occurred during the collection, handling, transport, and storage of snow samples. Precision and accuracy of Hg measurements in snow samples were determined based on standard deviation and the mean value of triplicate analysis of spiked standard solutions. The relative percentage of differences (RSD) for samples did not exceed 5 %. The standard stock solution of 1000 mg L^−1^ Hg in HNO_3_ was used to prepare five sub-standards 0.1, 0.5, 1.0, 2.5, and 5 ng (1.0, 5.0, 10.0, 25.0 and 50.0 ng L^−1^, respectively) for a multipoint calibration procedure (R^2^ > 0.998). The limit of detection, as three times the standard deviation of 10 blank samples, was about 0.1 ng L^−1^. Additionally, river water reference material with a certified mercury level of 12.6 ± 1.1 pg g^−1^ was used as an external standard (ORMS-3, National Research Council Canada). Method recovery was 97 ± 5 %.

Aliquots of filtered (0.45 μm Sartorius) snow samples were analyzed by ion chromatography (Dionex DX120, USA) for major water soluble anions: F^−^, Cl^−^, NO_3_^−^, and SO_4_^2−^, and cations: Na^+^, NH_4_^+^, K^+^, Mg^2+^, and Ca^2+^. Conductometric detection was coupled with post-column suppression of eluent ions. The detection limit of the analytical technique was calculated as three times the standard deviation (3*σ*) of a near-zero concentration measurements. The pH and electric conductivity (EC) values in all samples were determined at 25 °C, using a portable instrument SevenGo Duo (Mettler Toledo, UK) equipped with an automatic temperature compensation system. The average pH value was 4.8, and conductivity varied from 4.8 to 79.2 μS cm^−1^.

Total snowpack thickness corresponded to the depth of snow that was accumulated during a single precipitation event. In Poznań, snow cover thickness varied from 6 to 25 cm and was significantly lower and less stable compared with the polar snowpack.

### Statistical data analysis

To determine differences in concentrations between collected snow samples, data were checked for normality, outliers, and distribution, using Statistica 10.0 software. Pearson’s correlation (*p* value < 0.05) was used to establish statistically significant correlations between Hg and major ions in snow samples. A multivariate statistical method FA (Factor Analysis) was used to identify main factors, i.e., sources and processes controlling Hg snow chemistry. Basic meteorological parameters (precipitation, temperature, atmospheric pressure, relative humidity, wind speed, and direction) were provided by the local weather station at the Botanic Garden in Poznań. The regional/macro-regional potential emission sources of Hg were investigated with 4-day air-mass backward trajectories, generated using archive GDAS meteorological database (spatial resolution 1°), with HYSPLIT NOAA model (Draxler and Rolph 2013). Three starting heights of air masses, i.e., 500, 1000, and 1500 m were examined, roughly corresponding to daily height of the urban planetary boundary layer.

## Results and discussion

### Mercury and major ions in the snow cover from Poznań

Table 2 shows descriptive statistics for Hg, major ions, pH, and EC in shallow snow cover collected between January and March, 2013, in Poznań. The mean Hg concentration was 4.62 ng L^−1^ (±3.12), with maximum of 12.5 ng L^−1^. In 90 % of samples of the freshly fallen snow, Hg concentration was in the range between 0.98 and 10.5 ng L^−1^.

**Table 2:** Statistical summary of Hg, major ion concentrations, pH, and EC values, determined in snow samples from Poznań, Poland. Concentrations are given in nanogram per liter (Hg) and milligram per liter (all ions). Abbreviations are as follows: *MDL* method detection limit, *N* number of samples, *Q1* lower quartile, *Q3* upper quartile, percentile 5 and 95. Data were normally distributed

Species	Avg ± SD	Min	Max	Median	Q1	Q3	5 %	95 %
Hg	4.62 ± 3.12	0.43	12.5	3.43	2.48	6.84	0.98	10.55
F	0.02 ± 0.01	<MDL	0.03	0.02	0.02	0.02	0.01	0.03
Cl	0.73 ± 0.48	0.04	2.55	0.68	0.41	0.96	0.16	1.64
NO3	1.96 ± 1.49	0.06	7.75	1.55	1.07	2.27	0.65	5.18
SO4	1.24 ± 1.23	0.05	6.35	0.85	0.43	1.58	0.11	3.32
Na	0.11 ± 0.09	<MDL	0.46	0.08	0.04	0.16	0.02	0.31
NH4	0.50 ± 0.41	0.03	1.96	0.43	0.24	0.62	0.04	1.43
K	0.46 ± 0.47	<MDL	2.52	0.36	0.05	0.67	0.02	1.31
Mg	0.03 ± 0.04	<MDL	0.26	0.02	0.01	0.04	0.01	0.10
Ca	0.28 ± 0.31	0.03	2.29	0.23	0.09	0.34	0.04	0.72
pH	4.80 ± 0.41	3.39	6.12	4.78	4.51	5.02	4.12	5.50

Mean values of Cl^−^ and SO_4_^2−^ were 0.73 and 1.24 mg L^−1^, respectively. For these ions, the lower quartile was quite similar (Cl^−^ and SO_4_^2−^, 0.41 and 0.43 mg L^−1^), but the upper one was statistically different (Table 2). For the other inorganic ions, the concentrations ranged between 0.01 mg L^−1^ (F^−^, K^+^, Na^+^, Mg^2+^) and 7.75 mg L^−1^ (NO_3_^−^). The median values for the measured ions were in the following order: NO_3_^−^ > SO_4_^2−^ > Cl^−^ > F^−^ and NH_4_^+^ > K^+^ > Ca^2+^ > Na^+^ > Mg^2+^ for anions and cations, respectively. The pH value of snow samples from Poznań varied from 3.93 to 6.12, with the mean of 4.80 (±0.40), which was higher than for other urban/industrial sites characterized by elevated concentrations of air pollutants, i.e., PM_10_, PM_2.5_, PM_1_, CO, BC, NO_x_, NO, and SO_2_ (EEP report 2015). The mean electric conductivity (EC) was 20.69 ± 14.01 μS cm^−1^, and the majority of snow samples (90 %) had EC between 7.07 and 44.9 μS cm^−1^.

### Mercury in snow cover—comparison with other sites

The comparison of Hg concentrations in snow cover from northern high- and mid-latitude regions is shown in Table 3. Total mercury concentration measured in shallow snow cover from Poznań was significantly lower than in the Canadian Arctic during the AMDEs, i.e., in Alert (121 ng L^−1^, Steffen et al. 2002) and in Resolute (156 ng L^−1^, Lu et al. 2001).

**Table 3:** Comparison of mean concentrations of total mercury (ng L^−1^) in snow cover from different locations

Site	Site description	Hg concentration range (mean)	Reference
Poznań, Poland	semi-urban	0.43–12.49 (4.62)	This study
Gdynia, Poland	coastal/urban	2.7–22.5 (8.6)	Siudek et al. 2014
Alp, France	mountain	13–130	Ferrari et al. 2002
Col de Porte, France	mountain	80–160	Faïn et al. 2007
Alert, Canada	remote Arctic, tundra	5–121	Steffen et al. 2002
Acadia, USA	mountain/forest	2.8–37.0 (17.4)	Nelson et al. 2007
Siberian Altai, Russia	mountain glaciers	0.8–1.4	Eyrikh et al. 2003
Alps, Switzerland		1.5–2.0	
Tibetan Plateau, China	glacier snow	<1–43.6	Zhang et al. 2012
Hudson Bay, USA	sub-arctic	4–15.4 (10.6)	Dommergue et al. 2003
Mt. Oxford Icefield Agassiz Ice Cap	glacier, high Arctic	(0.38)	Zheng et al. 2014
		(0.66)	

In the present study, surface snow cover was not affected by AMDEs, however characterized by relatively small inter-seasonal variation of Hg compared to remote polar sites. In Poznań, distribution of Hg in snow cover was mainly determined by a local and regional anthropogenic sources (more intense during winter than in other seasons), meteorological situation and long-range transport of pollutants. Mean total Hg concentrations in the snowpack from French mountain stations, i.e., Alp (Ferrari et al. 2002) and Col de Porte (Faïn et al. 2007) were much higher than in Poznań (Table 3). The observations of extremely high Hg amount in alpine snow samples were linked to local urban plume episodes and atmospheric conditions that enhanced Hg retention in the snowpack. The Hg concentration range for snow samples from these sites was also higher than for samples from Acadia watersheds in Maine, US (Nelson et al. 2007), where the minimum Hg value was 2.8 ng L^−1^ and the maximum was 37.0 ng L^−1^. The maximum value of Hg from our study showed good agreement with the values from sub-arctic sites, i.e., Hudson Bay region (15.4 ng L^−1^, Dommergue et al. 2003). The mean Hg concentrations in snow cover from the semi-urban site in Poznań were much higher than in snow cover from Mt. Oxford Icefield (0.38 ng L^−1^) and Agassiz Ice Cap (0.66 ng L^−1^)—high Arctic regions (Zheng et al. 2014). There are also several other Hg measurements in snow pit from high-altitude sites in Siberian Altai and Swiss Alps (Eyrikh et al. 2003). All these studies showed relatively lower Hg concentrations in snowpack than those measured in Poznań, with maximum Hg concentration in snow samples below 2.0 ng L^−1^. In contrast, elevated concentrations of total Hg were observed in glaciers snow from Tibetan Plateau, western China (Zhang et al. 2012) mainly as a result of high atmospheric Hg loading from regional and global sources. These studies also showed significant seasonal variations of Hg in snow pits that were much lower in summer than in winter (Zhang et al. 2012).

### Hg and other ions’ origin in snow cover

Factor analysis (FA), an orthogonal transformation method with normalized varimax rotation, was applied to the log-transformed database of 12 variables (H^+^, EC, total Hg, F^−^, Cl^−^, NO_3_^−^, SO_4_^2−^, Na^+^, NH_4_^+^, K^+^, Mg^2+^, Ca^2+^), to determine principal components with eigenvalues greater than 1. The selected factors were then attributed to the main atmospheric processes and sources of pollutants accumulated in the snow cover from Poznań during winter 2013. A three-factor solution of FA explained 73 % of the total variance (Table 4).

**Table 4:** Factor analysis of inorganic cations, anions, total Hg, and electric conductivity measured in snow cover, Poznań. All variables from the database were log-transformed before mathematical calculations

Variables	Factor loading		
	FA1	FA2	FA3
H^+^	*0.82*	−0.28	0.05
EC	*0.75*	−0.24	0.11
Hg	0.06	0.00	−*0.75*
F	−0.49	0.37	−0.28
Cl	0.06	*0.87*	−0.08
NO_3_	*0.81*	0.28	0.17
SO_4_	*0.89*	−0.04	0.00
Na	0.51	0.27	*0.65*
NH_4_	*0.84*	0.18	−0.15
K	−0.21	*0.87*	−0.11
Mg	0.05	*0.81*	0.47
Ca	−0.01	*0.77*	0.45
Eigenvalue	4.08	3.43	1.22
Percent of variance	34	29	10
Cumulative percentage	34	67	73

Parameters with high loading (>0.6) were set in italics

Factor FA1 (acidifying pollutants: H^+^, NO_3_^−^, SO_4_^2−^, Na^+^, NH_4_^+^, and EC) was a major component of the overall variance (34 %, Table 4). Factor FA2 indicated four statistically significant variables and contributed 29 % to the total variance. Two industrial pollutants, i.e., Ca^2+^ and Mg^2+^, with high loadings of 0.77 and 0.81, pointed to the transport of fine and coarse particles from different anthropogenic sources, such as construction dust, coal combustion, vehicle emission, and road dust. FA2 factor was also explained by high loading of Cl^−^ and K^+^ (0.87). The obtained values suggest strong contribution of acidic inorganic salts from transportation sources (dust, resuspension, traffic) during snow episodes in Poznań. However, other local/regional industrial processes and long-range transport of pollutants cannot be ruled out.

The third factor (10 % of total variance) was characterized by two species with loadings higher than 0.6. Results from FA3 showed that total mercury had statistical significance and inverse correlation with Na^+^, suggesting some different sources or complex atmospheric processes in which these two components participated. Due to the fact that FA analysis cannot clearly explain the inter-seasonal variability of Hg in snow cover or Hg interactions with all variables, some additional data (e.g., meteorological, local/regional atmospheric transport models) and ion molar ratios (i.e., chloride to sulfate, nitrate to sulfate, sulfate to sodium) are needed to assess the role of atmospheric conditions and importance of anthropogenic emission during snow events. These aspects are discussed in the next section.

### Vertical distribution of Hg in snow cover

**Fig. 2:**
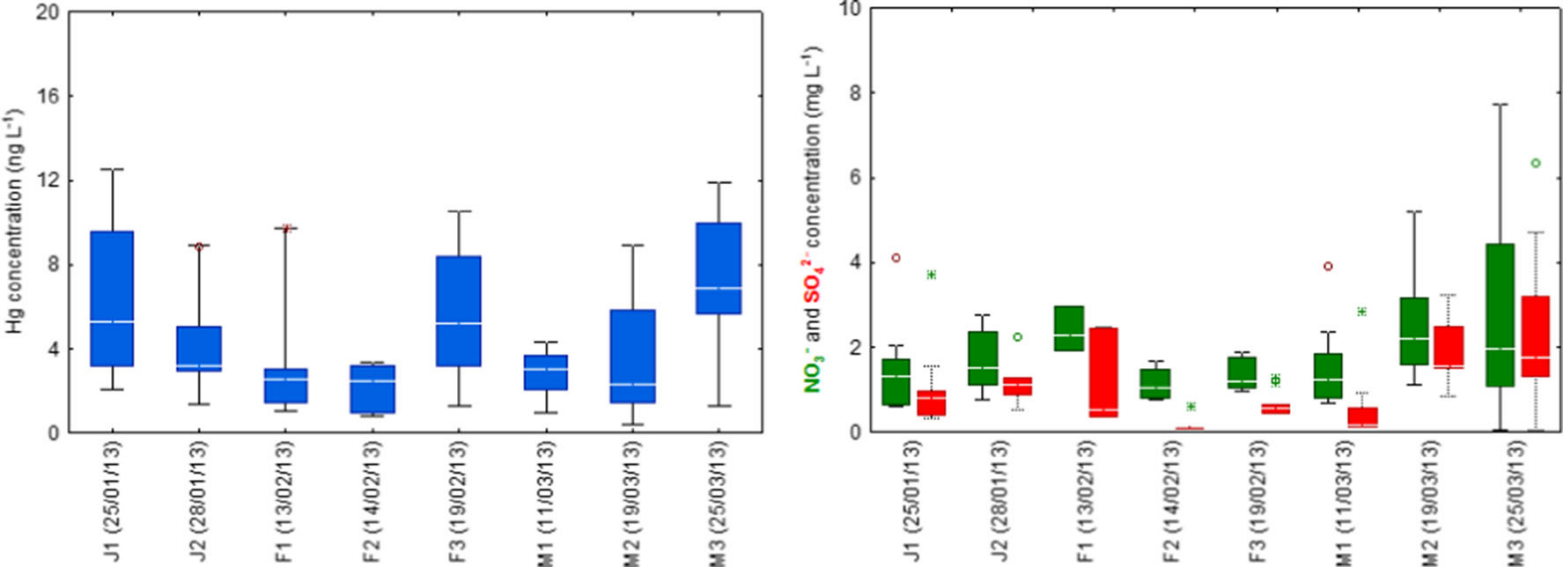
Box plot of total Hg (*left*) and NO_3_^−^/SO_4_^2−^ (*right*) concentrations in the snowpack. The inter-seasonal variation in Hg concentrations was not statistically significant (Kruskal-Wallis test, *p* value < 0.05). The *horizontal lines* represent median, the *box spans*—the quartiles, and the *whiskers* indicate the maximum and minimum values

Figure 2 illustrates the variation in concentration of total Hg and ions (NO_3_^−^ and SO_4_^2−^) in the snow cover from Poznań. In general, peak concentration of Hg and major ions did not occur during the same snow episode, suggesting large variability in chemical composition, impact of different industrial/urban sources, air mass transport, and fluctuations in atmospheric conditions, in particular air temperature and wind speed/direction. High variability in Hg concentration was found for most snow samples (J1 to J3, F2, M2, and M3).The lowest value occurred in March (M2).

So far, several continuous observations have been undertaken to examine inter-seasonal variability of Hg in well-stratified polar snowpack (Douglas et al. 2008, Dommergue et al. 2010) and shallow snow cover from mid-latitudes (Siudek et al. 2014). Recent studies carried out in polar environments have documented that Hg distribution pattern within the snowpack is affected by complex physical processes and heterogeneous chemical reactions with photo-labile halogens such as bromine, bromine monoxide, chlorine, and chlorine monoxide, and is controlled by different meteorological parameters (Steffen et al. 2015). Among these variables, the most important for Hg retention in snow and/or Hg re-emission through air-snow transition layer are cloudiness, air temperature and relative humidity, height of mixing layer, wind speed, solar radiation, snow composition, and type of sites (Johnson et al. 2008, Nelson et al. 2010, Faïn et al. 2013, Angot et al. 2016).

**Fig. 3:**
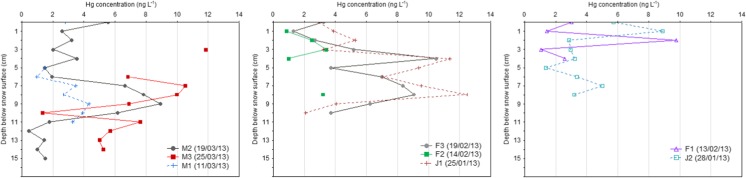
Vertical profiles of total Hg concentration (ng L^−1^) in snow cover. Snow experiments were grouped in three sections based on snow cover thickness. It was as follows: *left panel* (samples with the largest depth), *middle* (samples with medium depth), and *right panel* (samples with the lowest depth)

For polluted mid-latitude environments, the most important factors influencing Hg deposition are the winter maxima of anthropogenic emission and local meteorology. This was observed in Poznań, where vertical concentration profiles of Hg were very irregular and without general trends during all snow experiments. The distribution of Hg values measured in snow cover is shown in Fig. 3.

Different profiles of total Hg concentration obtained in this study may be linked to specific small-scale processes/sources (mainly local coal combustion) and fast changing atmospheric conditions. For example, the precipitation event on 19 March 2013 (snow depth = 25 cm) was associated with very unstable atmospheric conditions, i.e., convective clouds, large gradient of air temperature −4.7 to 4.5 °C, air pressure 1002–1007 hPa, and wind velocity 1.1 to 3.7 m/s (Table 1). In this case, Hg concentration correlated well with NO_3_^−^ (0.537, *p* value = 0.351), and the mean NH_4_^+^/SO_4_^2−^ molar ratio was 0.99. However, Hg did not show positive correlation with SO_4_^2−^, and the SO_4_^2−^/NO_3_^−^ molar ratio was 0.31. This suggests lower influence of local SO_2_ emission on snow chemistry. Filippa et al. (2010) and Puxbaum and Wagenbach (1994) reported higher molar ratios of sulfate-to-nitrate in alpine snowpack, indicating significant impact of continental pollution and specific role of orographic clouds. Nitrate, a marker of anthropogenic emission that typically increases during winter season with decreasing mixing layer height, was a dominant ion in the snowpack from Poznań. Its concentration ranged between 0.68 and 5.18 mg L^−1^. The above-mentioned snow episode (19 March 2013) reflects the importance of aqueous/particle-phase reactions of Hg with acidifying compounds during the below-cloud processes or inside the snowpack after deposition. Moreover, relatively high wind variability, from the southeast to south (Table 1), over a short timescale, explained the predominant contribution of local and regional emissions (i.e., CFFPs Poznań Karolin, metallurgical factory, residential boilers, waste incinerations, rail freight transport, and road traffic). Furthermore, cluster analysis of backward trajectories indicated air masses with pollutants mainly from central and southeastern European industrial hot spots. In contrast to this case, the mean concentration level of Hg above 5 ng L^−1^, measured in the snowpack on 25/01 (J1), 19/02 (F3), and 25/03 (M3), was generally attributed to air mass transport from the north-east to north-west (Fig. 2). In addition, during these measurements, wind speed was low (on average < 1.5 m/s, Table 1), indicating relatively inefficient dispersion conditions, probably much more prone to stimulate self-coagulation processes of atmospheric Hg-enriched particles and its deposition with snowfall.

The differences between examples of snow episodes from southern sector (M2 vs. F2) might be partially explained by larger contribution from local fossil fuel combustion (commercial and domestic heating) in samples associated with S-SE airflow compared to the cases attributed to SE-E wind direction. The SE-E areas surrounding Poznań are characterized by significantly lower density of residential buildings and lower influence of local industrial emission and transport. Furthermore, with reference to Table 1, mean air temperature during 14/02 (F2) and 11/03 (M1) snowfalls was −0.4 and −2.9 °C, respectively, which was lower than on 13/02 and 19/03 (F1 1.1 °C and M2 0.1 °C, respectively). The measurements by Enroth et al. (2016) showed that under low temperature, urban aerosol dynamics can be widely controlled by two main processes, i.e., nucleation and condensation. Therefore, the observed non-linear shape of Hg and major ion concentrations in snow cover from Poznań can be regarded as an important proxy for studying changes in atmospheric composition (air quality index, aerosol loading, partitioning processes, sources contribution) and transport (dilution effect, deposition, etc.).

Based on polar snow experiments, it was found that formation of Hg° in the interstitial air inside the snowpack can be mainly initiated by photochemical reduction of divalent mercury species, catalyzed by halogen anions (Br, Cl) and high level of oxidizing species (Faïn et al. 2007). As expected, due to low or near-zero UV radiation from January to March in Poznań, no statistically significant re-emission of gaseous Hg° from the snowpack to the overlaying atmosphere was observed for the whole snow season. Similar findings were reported by Siudek et al. (2014) for the single coastal site in Gdynia (northern Poland). The study carried out in Gdynia demonstrated preferential mechanism of Hg retention within the snowpack and direct transfer of Hg to the soil system during melt season. In addition, high variability of Hg concentrations in meltwater, ranging from 0.6 to 10.8 ng L^−1^, with the average value of 4.4 ng L^−1^, indicated that the significant load of mercury per meter square was retained in the snowpack (Siudek et al. 2014). In contrast, in the present study, we found less variable Hg concentrations measured in meltwater, which could be linked to substantially lower contribution from local anthropogenic sources observed at this semi-urban station as compared to the coastal site during winter season. Our findings are also consistent with observations from forested and no-canopy sites in the US (Nelson et al. 2008), where large Hg retention was the main pathway of post-depositional processes of Hg within the snowpack.

### Atmospheric long-range transport of mercury

**Fig. 4:**
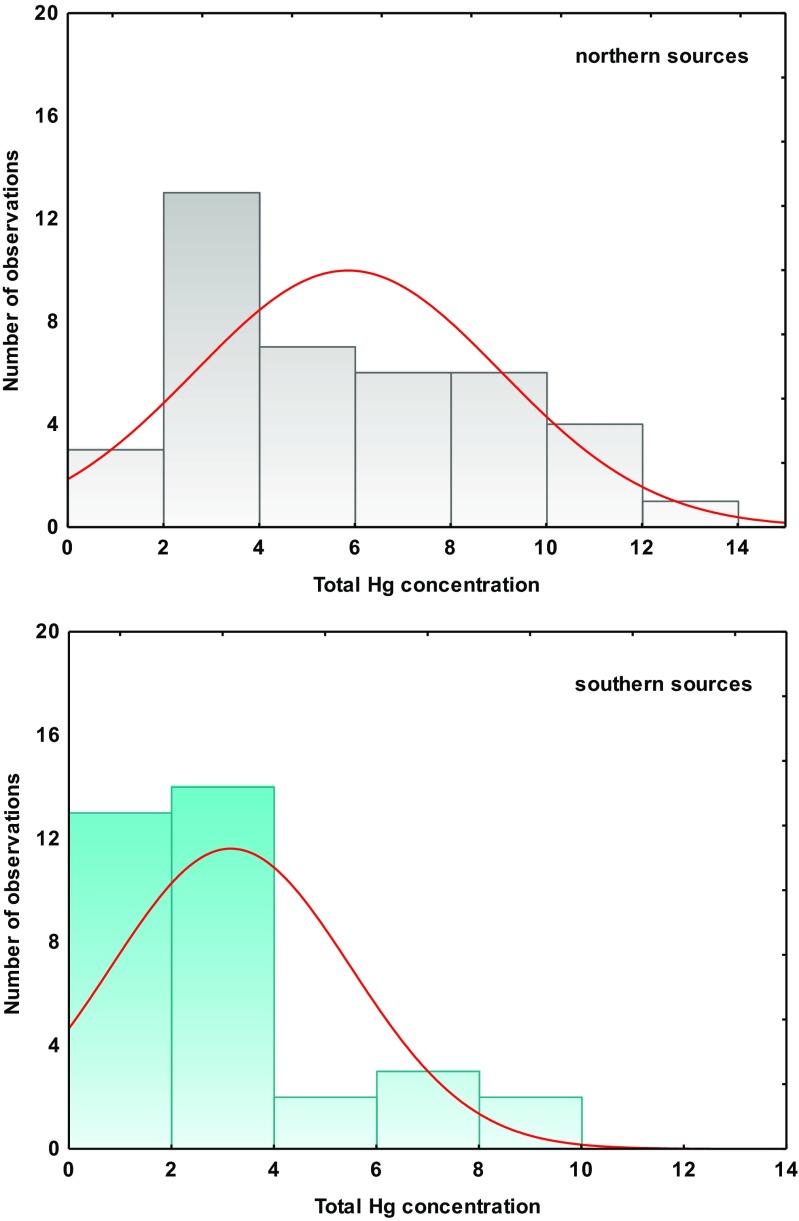
Frequency distribution of total mercury concentration measured in snow samples associated with **a** northern **b** southern advection during measurements in January–March 2013, Poznań

Figure 4 presents histograms of total Hg concentration in snow samples as a function of wind sector. Specifically, differences between samples attributed to northerly (N-NE, NW-N, W-NW, NE-E) or southerly (SE-S, SE-E) advection were pronounced for the concentration threshold of 4 ng Hg L^−1^. In the first backward trajectory cluster, representing mainly northern sources (snow episodes defined as J1, J2, F3, M1, M3, see Table 1), 62 % of values ranged from 4 to 14 ng L^−1^, whereas for the snow cover associated with southern advection (snow episodes: F1, F2, M2), the histogram was shifted towards lower concentrations (>MDL to 4 ng L^−1^), which did not exceed 10 ng L^−1^ (Fig. 4). The effect of advection on Hg concentration in snow samples explains most of its variability and shows large influence of various local/regional anthropogenic sources. Siudek et al. (2015b) have previously studied distribution of trace metals such as Cu, Cr, Cd, Pb, Ni, As, and Zn in snowpack, based on backward trajectory simulations. For northerly and northeasterly airflow, concentrations of Pb, Zn, and Cr in snow cover were higher. Therefore, the variation in Hg concentration in freshly fallen snow samples collected after the predominant N to NE advection of polluted air masses was probably caused by atmospheric processes and high contribution from the similar sources as in the case of Pb, Zn, and Cr (i.e., fossil fuel combustion, traffic-related emission, processing of non-ferrous metals).

**Fig. 5:**
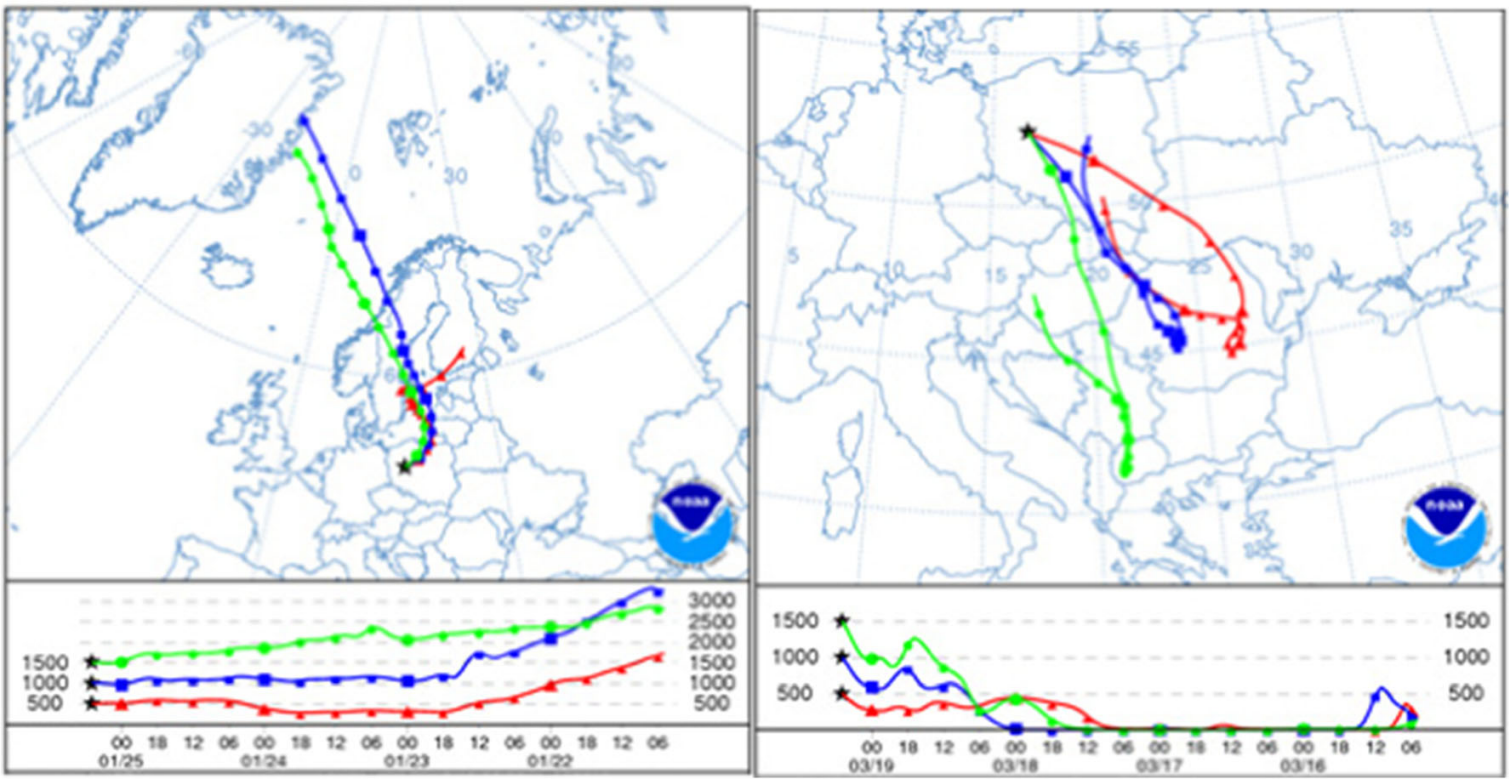
An example of 4-day backward trajectories for snow cover collected in Poznań on **a** 25 January 2013 (*left*) **b** 19 March 2013 (*right*). Starting heights of backward trajectories were 500 m (*triangles*), 1000 m (*squares*), and 1500 m (*circles*)

In order to better estimate the effect of long-range transport on high Hg content in the snowpack from Poznań, we examined two clusters representing different types of air masses which transported pollutants over the study domain from south or north directions. Here, we focused specifically on two snow episodes that were representative for each wind sector (Fig. 5).

The first case of below-cloud scavenging of Hg species by snow was observed on 25 January 2013 (Fig. 5a). It should be highlighted that the above-mentioned snow episode was the first precipitation event after a relatively long period without rain-/snowfalls. A 4-day backward trajectory showed the predominance of northerly and northeasterly airflow regime with large precipitation, which revealed major source areas for pollutants accumulated in snow cover in Poznań. They were as follows (in increasing order of importance): northern high latitudes (Greenland, North Atlantic), Scandinavian Peninsula, Baltic Sea, Russia, Lithuania, and northeastern Poland. Due to the fact that urban/industrial emission from polar regions and northern European countries is rather low as compared to central/western Europe, the northern regions had probably minor contribution to the total Hg pool in snow cover in Poznań. In addition, relatively low wind speeds on 25 January, from 0.1 to 1.1 m/s, suggested local and regional transport towards the sampling site, from such sources as residential boilers, domestic heating units, fossil fuel combustion in commercial sector, military area in Biedrusko, and waste incineration. Moreover, nocturnal inversion layer might have favored the accumulation of air pollutants and led to higher concentrations of Hg in the lower atmosphere over the study region just before the J1 snow experiment. The air temperature during those days was also very low (ranging from −12.4 to −0.2 °C), causing an intensified residential fuel consumption.

The second case was associated with the snow experiment on 19 March (M2), with quite different atmospheric conditions, i.e., air temperature between −4.7 and 4.5 °C, wind speed from 1.1 to 3.7 m/s (Table 1). During this experiment, a predominant south-eastern to southern advection was observed. Hence, urban plumes from local combustion processes, with minor contribution from long-range transport, were the most probable explanation for elevated Hg concentrations measured in snow cover. For comparison, the same airflow pattern was observed during F1 experiment (Table 1). However, Hg concentration was slightly higher compared to the case of 19 March. It is important that both snow covers did not include ice layers that might have prevented upward migration of Hg species through the snowpack, which would greatly affect its chemistry. In addition, snow cover that was formed during F1 experiment was much thicker than that from M2 experiment. Thus, it was not a surprise to notice much quicker Hg transfer to soil/ground after melting. Nevertheless, the observed differences in Hg content in snow cover in Poznań were caused not only by inter-seasonal variation in pollutant loadings (residential and commercial sector), but also by a combination of atmospheric chemistry and dynamics.

It can be seen that low temperatures together with the increase in relative humidity largely enhanced Hg transformation processes in the lower atmosphere, including coagulation and sorption of compounds onto airborne particles (Humphries et al. 2015). Furthermore, such a temperature-induced mechanism has fundamental impact while considering partitioning between soluble and particulate phase of Hg (Rutter and Schauer 2007). Several recent studies revealed that the composition of urban aerosol strongly affects vertical profiles of atmospherically deposited Hg inside the snow cover. However, further observations in urban areas are still needed to provide underpinning data for better understating the role of thick and very unstable snow cover in Hg chemistry at mid-latitude sites.

## Conclusions

In this study, chemical composition of snow cover was investigated and the results of 3-month field measurements at the semi-urban site in Poznań were thoroughly examined. The applied sampling method was a novel approach and an important criterion for quantitative determination of Hg in snow cover.

Our results clearly indicated the importance of Hg measurements in a shallow snow cover of industrially impacted areas in the estimation of local and regional-scale Hg budget. The inter-seasonal variability of Hg concentrations in snow cover was mainly driven by two factors: seasonal changes in meteorological conditions (especially fluctuations in ambient temperature and wind pattern) and seasonal variation in Hg emission from local/regional anthropogenic sources (coal combustion). Based on vertical profiles of Hg concentration in snow cover, it was found that mercury had relatively irregular distribution, affected by high pollution events due to intensive coal combustion in the study area. In January 2013, we observed a peak in Hg concentrations in snow cover, suggesting large contribution from major anthropogenic point sources located in the vicinity of the study area.
